# The NAE inhibitor pevonedistat (MLN4924) synergizes with TNF-*α* to activate apoptosis

**DOI:** 10.1038/cddiscovery.2015.34

**Published:** 2015-10-05

**Authors:** F S Wolenski, C D Fisher, T Sano, S D Wyllie, L A Cicia, M J Gallacher, R A Baker, P J Kirby, J J Senn

**Affiliations:** 1 Drug Safety Research and Evaluation, Millennium Pharmaceuticals, Inc., a wholly owned subsidiary of Takeda Pharmaceutical Company Limited, 35 Landsdowne Street, Cambridge, MA, USA; 2 Drug Safety Research Laboratories, Takeda Pharmaceutical Company Limited, 26-1 Muraoka-Higashi 2-chome, Fujisawa, Kanagawa, Japan

## Abstract

Predicting and understanding the mechanism of drug-induced toxicity is one of the primary goals of drug development. It has been hypothesized that inflammation may have a synergistic role in this process. Cell-based models provide an easily manipulated system to investigate this type of drug toxicity. Several groups have attempted to reproduce *in vivo* toxicity with combination treatment of pharmacological agents and inflammatory cytokines. Through this approach, synergistic cytotoxicity between the investigational agent pevonedistat (MLN4924) and TNF-*α* was identified. Pevonedistat is an inhibitor of the NEDD8-activating enzyme (NAE). Inhibition of NAE prevents activation of cullin-RING ligases, which are critical for proteasome-mediated protein degradation. TNF-*α* is a cytokine that is involved in inflammatory responses and cell death, among other biological functions. Treatment of cultured cells with the combination of pevonedistat and TNF-*α*, but not as single agents, resulted in rapid cell death. This cell death was determined to be mediated by caspase-8. Interestingly, the combination treatment of pevonedistat and TNF-*α* also caused an accumulation of the p10 protease subunit of caspase-8 that was not observed with cytotoxic doses of TNF-*α*. Under conditions where apoptosis was blocked, the mechanism of death switched to necroptosis. Trimerized MLKL was verified as a biomarker of necroptotic cell death. The synergistic toxicity of pevonedistat and elevated TNF-*α* was also demonstrated by *in vivo* rat studies. Only the combination treatment resulted in elevated serum markers of liver damage and single-cell hepatocyte necrosis. Taken together, the results of this work have characterized a novel synergistic toxicity driven by pevonedistat and TNF-*α*.

## Introduction

Inflammation can be a driver of drug toxicity.^1^ Molecular mechanisms of toxicity have been characterized in cell-based models through the combination of treatment with inflammatory cytokines and pharmacological agents of interest.^2,3^ This approach has also been utilized in animal models of adverse drug.^4^ The common goal of these *in vitro* and *in vivo* models is to identify and characterize the molecular mechanisms that drive toxicity. We present a model describing a synergistic cytotoxicity between pevonedistat and TNF-*α*. The *in vitro* mechanism of death was caspase-8-mediated apoptosis. This synergistic cytotoxicity was also identified in a rat model, in which the combination treatment of pevonedistat and elevated TNF-*α* resulted in liver damage.

The investigational compound pevonedistat (MLN4924) is a small-molecule inhibitor of NEDD8-activating enzyme (NAE) that has been evaluated in clinical trials for the treatment of acute myelogenous leukemia,^5,6^ myelodysplastic syndrome,^5^ solid tumors,^7,8^ nonhematological malignancies,^9^ melanoma,^10^ lymphoma, and multiple myeloma.^11^ The role of NAE is to transfer NEDD8, an ubiquitin-like protein, to downstream substrates such as cullin-RING ligases (CRLs) via the NEDD8-conjugating enzyme UBC12.^12^ The CRL–NEDD8 complexes function as ubiquitin ligases that attach ubiquitin to substrates to target them for proteasome degradation.^13^ Inhibition of NAE by pevonedistat ultimately leads to CRL inactivation and accumulation of various substrates normally degraded via the ubiquitination pathway.^14^ Pevonedistat causes cell death through DNA re-replication and cell-cycle arrest that results from accumulation of the CRL substrate CDT1.^14,15^ Additionally, pevonedistat can block pro-survival NF-*κ*B signaling by preventing the degradation of phospho-I*κ*B*α*.^16^ In a phase 1 trial, a subset of patients treated with high doses of pevonedistat experienced adverse events that included elevated hepatic transaminases and multi-organ failure following the first dose of pevonedistat.^5^ These findings served as the impetus for developing a preclinical model of pevonedistat drug-induced liver toxicity.

The pro-inflammatory cytokine TNF-*α* is not only critical for innate immune function but also has a role in inflammatory responses.^17^ TNF-receptor (TNF-R) binds TNF-*α* that along with TRAIL-R (DR4/DR5) and Fas-R (CD95) comprise the family of death receptors.^18,19^ Initiation of TNF signaling leads to either pro-survival (NF-*κ*B and JNK) or pro-death (caspase-3 and -8) outcomes.^20^ Activation of apoptosis is tightly regulated and requires posttranslational modifications of numerous proteins. A critical step is the activation of caspase-8 through a series of cleavages from pro-enzyme to active protease.^21,22^ The two activated protease domains of caspase-8, p18 and p10, cleave downstream substrates such as caspase-3 to propagate apoptosis.^23,24^ The active form of caspase-8 is composed of a heterotetramer consisting of p18_2_/p10_2_.^18,25^ Death receptor signaling can also drive a related, although mechanistically distinct, cell-death pathway called necroptosis.^26,27^ Activation of this pathway requires the caspase-8 protein to remain in its uncleaved pro-enzyme form.^28^ This leads to the formation of the necroptosome (RIP1, RIP3, trimeric MLKL) that ultimately kills cells through loss of mitochondria membrane potential.^29–31^

The results of this study describe a liver model for pevonedistat drug-induced toxicity that is dependent on co-treatment with the inflammatory TNF-*α* cytokine. Additionally, pevonedistat proved useful as a tool compound for the molecular characterization of cell-death pathways.

## Results

### Pevonedistat+TNF-*α* is cytotoxic *in vitro* to cultured cells

**Figure 1:**
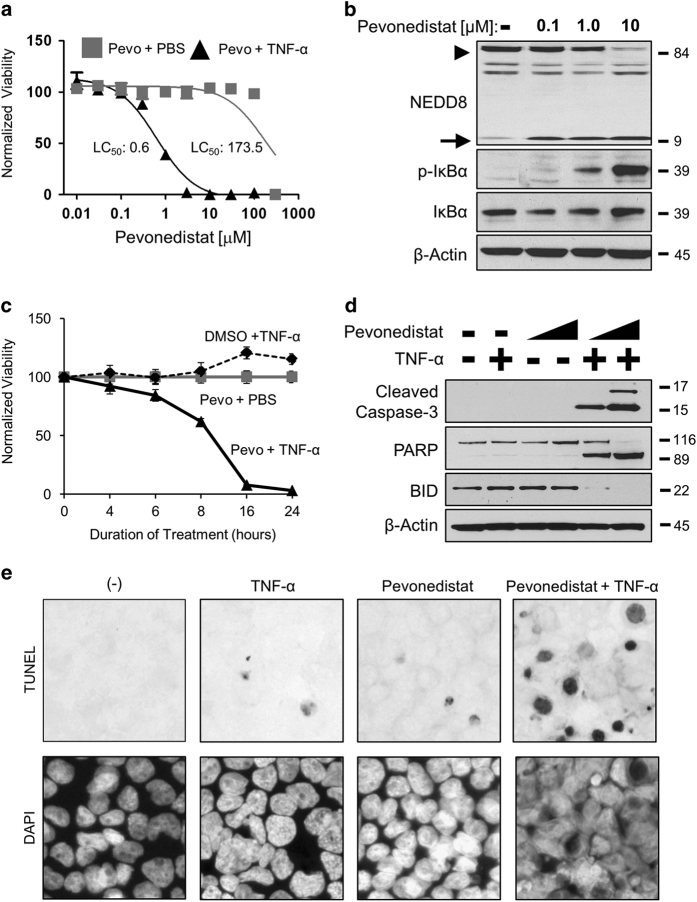
Pevonedistat+TNF-*α* is cytotoxic. (**a**) Cultured rat H-4-II-E cells were treated with pevonedistat in combination with either PBS (gray boxes) or 5 ng/ml TNF-*α* (black triangles) for 24 h. The LC_50_ was determined using the least-squares method, and solid lines indicate a non-linear fit of the data. (**b**) Cells were treated with DMSO (−) or pevonedistat (0.1, 1.0, and 10 *μ*M) for 8 h. Lysates were western blotted for the indicated proteins. NEDD8-cullin (arrowhead) and unbound NEDD8 (arrow) are indicated (**c**). Viabilities were determined at the indicated time points after treatment with DMSO+5 ng/ml TNF-*α* (black circles), 10 *μ*M pevonedistat+PBS (gray boxes), or 10 *μ*M pevonedistat+5 ng/ml TNF-*α* (black triangles). (**d**) Lysates of cells treated with 1 or 10 *μ*M of pevonedistat±TNF-*α* for 8 h were western blotted for the indicated apoptotic marker proteins. (**e**) TUNEL (terminal deoxinucleotidyl transferase-mediated dUTP-fluorescein nick end labeling; upper) and DAPI (4,6-diamidino-2-phenylindole; lower) staining of apoptotic cells after 6 h of treatment. All viability experiments were performed in triplicate and error bars indicate±S.E.M. Approximate molecular sizes of proteins (in kDa) are given to the right of blots.

A synergistic cytotoxicity was identified between pevonedistat and TNF-*α* in the rat hepatoma H-4-II-E cell line. Comparison of the lethality for 50% of cells (LC_50_) indicated the combination of pevonedistat+TNF-*α* was approximately 300-fold more toxic than single-agent pevonedistat (Figure 1a). Knockdown of NEDD8 expression with siRNA, which mimicked the inhibitory effect of pevonedistat, also sensitized cells to TNF-*α* (Supplementary Figure S1). Western blotting of lysates from H-4-II-E cells treated with 10 *μ*M pevonedistat indicated the disappearance of a band corresponding to NEDD8-cullin (Figure 1b, arrowhead) with concurrent buildup of unbound NEDD8 (arrow). This concentration of pevonedistat also resulted in the accumulation of CRL substrate phospho-I*κ*B*α*, consistent with previous findings,^32^ and did not affect the expression of total I*κ*B*α*. Unless otherwise indicated, the concentrations of 10 *μ*M pevonedistat and 5 ng/ml TNF-*α* were used in subsequent *in vitro* experiments. Pevonedistat+TNF-*α* resulted in a rapid cell death, killing ~95% of cells within 16 h (Figure 1c). Three apoptosis markers (cleaved caspase-3, PARP, and BID) were only cleaved after the combination treatment (Figure 1d). Consistent with this result, a TUNEL assay for nicked DNA confirmed that the pevonedistat+TNF-*α* treatment resulted in more stained cells than any single-agent treatment (Figure 1e). The pevonedistat+TNF-*α* synergistic cytotoxicity was replicated in a diverse set of other cell types, including: primary rat hepatocytes and liver Kupffer cells; the rat proximal tubule line NRK-52E; the human acute monocytic leukemia line THP-1; and the human hepatocellular carcinoma line HEP-G2 (Supplementary Figure S2). Of note, THP-1 cells were sensitive to pevonedistat in combination with either the human TNF-*α* cytokine or with an agonist antibody to human TNF-R (Supplementary Figures S2d and e).

### DNA re-replication does not drive pevonedistat+TNF-*α* toxicity

**Figure 2:**
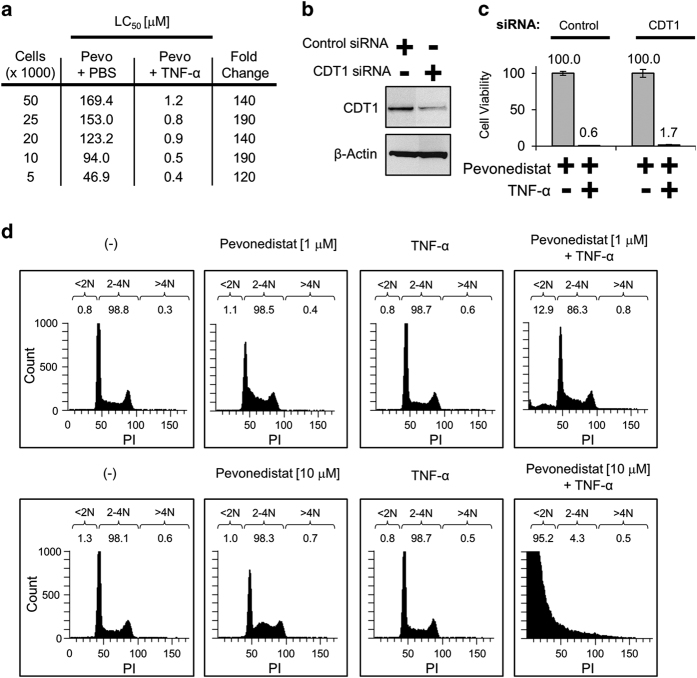
DNA re-replication does not drive pevonedistat+TNF-*α* toxicity. (**a**) H-4-II-E cells were seeded from sparse (5000) to confluent (50 000) in wells of a 96-well plate. Cells were treated with pevonedistat±TNF-*α*, and viabilities were determined after 24 h. (**b**) Cells were transfected with either a siRNA pool against a non-targeting control or a single oligonucleotide siRNA against CDT1. Lysates were collected 5 days later and western blotted for the indicated proteins. (**c**) Viabilities of cells transfected with control or CDT1 siRNAs treated with pevonedistat±TNF-*α* were determined after 48 h. Viability experiments were performed in triplicate and error bars indicate±SEM. (**d**) Actively dividing cells were treated with 1 *μ*M pevonedistat (upper) or 10 *μ*M pevonedistat (lower)±TNF-*α* for 8 h. Cells were then pulsed with Brd-U and fixed, and DNA content was determined via FACS analysis. Displayed are cells that stained positive for PI *versus* the count of Brd-U positive. DNA content was determined by sorting cells into <2*N*, 2–4*N*, and >4*N* groups and displayed as a percentage of the total live cells counted.

Single-agent pevonedistat prevents the degradation of the CRL substrate CDT1, which results in DNA re-replication, cell-cycle arrest, and ultimately cell death.^12–16^ Previous work demonstrated that actively dividing cells were the most sensitive to pevonedistat.^14^ In this study, H-4-II-E cells were seeded from sparse to confluent and treated with pevonedistat±TNF-*α*. The density of cells did not confer resistance to the combination treatment, suggesting that the cytotoxicity observed with combination treatment is independent of cellular replication state (Figure 2a). The role of CDT1 in this toxicity was then directly assessed with siRNA knockdown. An ~80% knockdown of CDT1 (Figure 2b) did not prevent death caused by pevonedistat+TNF-*α* (Figure 2c). The amount of DNA re-replication was quantified with FACS analysis. Cells received either a low (1 *μ*M) or high (10 *μ*M) dose of pevonedistat±TNF-*α*. After an 8 h incubation, the relative amount of >4*N* DNA indicative of re-replication was similar (<1%) among all treatments (Figure 2d). Of note, pevonedistat+TNF-*α* caused an accumulation of <2*N* DNA dead/fragmented cells that increased concentration dependently with pevonedistat (right boxes).

### Pevonedistat+TNF-*α* toxicity is mediated by caspase-8

**Figure 3:**
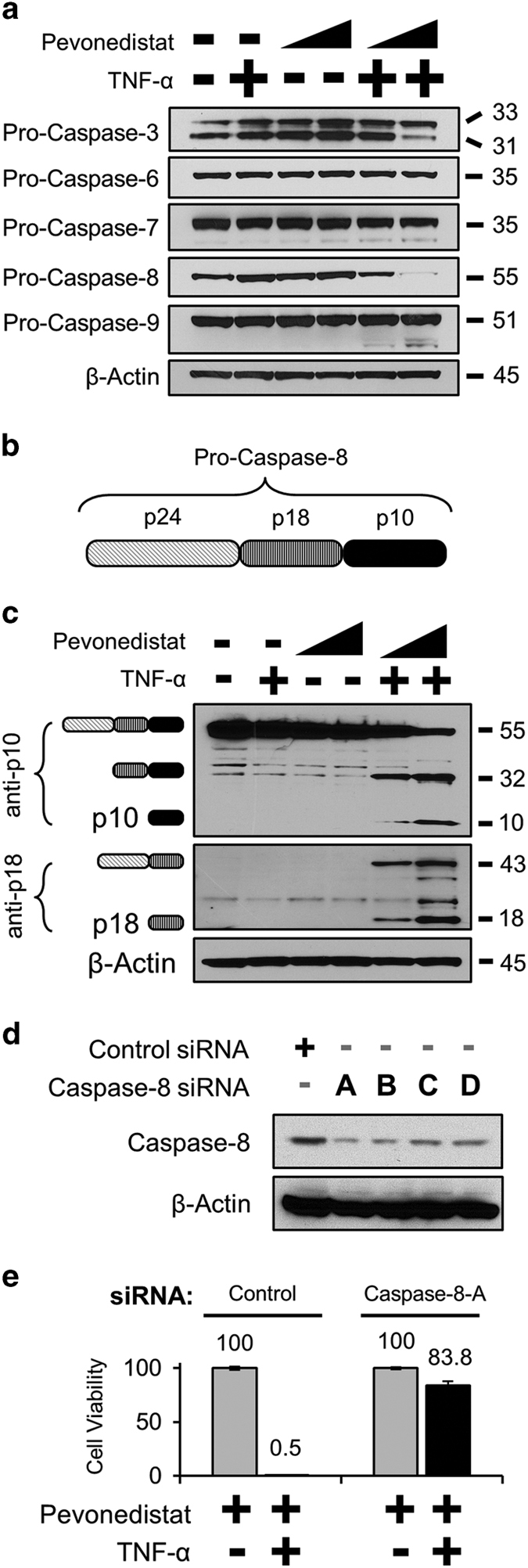
Pevonedistat+TNF-*α* cytotoxicity is mediated by caspase-8. (**a**) H-4-II-E cells were treated with 1 or 10 *μ*M pevonedistat±TNF-*α* for 16 h. Extracts were western blotted for the pro-enzyme form of the indicated caspases. (**b**) The schematic of the individual subunits of pro-caspase-8 (p24, p18, and p10) are as indicated. (**c**) Lysates from cells treated with 1 or 10 *μ*M pevonedistat±TNF-for 8 h were western blotted with antibodies specific for epitopes within the caspase-8 p10 (top) or p18 subunits. The predicted caspase-8 subunits are indicated to the left of the image based on the expected size of the product. (**d**) Lysates from cells transfected with siRNA oligonucleotides against either a non-targeting control or against caspase-8 were western blotted for full-length caspase-8. (**e**) Cells were transfected with either a non-targeting control or the caspase-8-A siRNA. Four days later, cells received the indicated treatments and viability was assessed after an additional 48 h. All viability experiments were performed in triplicate, and error bars indicate±S.E.M. Approximate molecular sizes of proteins (in kDa) are given to the right of blots.

Pro-caspase-8, and to a lesser extent pro-caspase-3, was cleaved/activated in cells treated with pevonedistat+TNF-*α* (Figure 3a). Pro-caspase-8 is comprised of three domains (Figure 3b), but only p18 and p10 are proteases.^21^ Two caspase-8 antibodies specific for different areas of the protein detected the numerous cleavage products that resulted from pevonedistat+TNF-*α* (Figure 3c). Knockdown of caspase-8 expression with siRNA was optimized with single oligonucleotides (Figure 3d). Compared with control cells treated with pevonedistat+TNF-*α* (~1% viability), the caspase-8-A siRNA knockdown cells tolerated the treatment (84% viability) over 48 h (Figure 3e). These results clearly demonstrate that caspase-8 mediates the synergistic cytotoxicity of pevonedistat+TNF-*α*.

**Figure 4:**
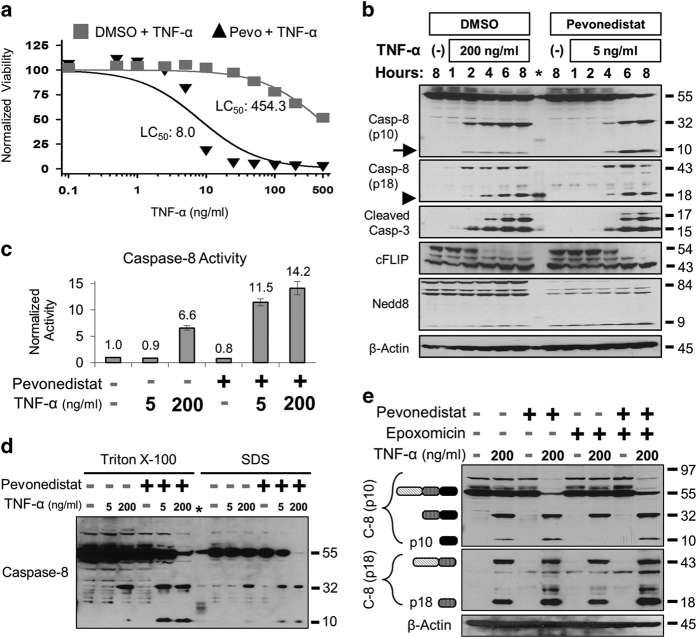
Pevonedistat+TNF-*α* drives caspase-8 activation and p10 accumulation. (**a**) H-4-II-E cells were treated for 8 h with TNF-*α* (doses ranging from 0.1 to 500 ng/ml) in combination with either vehicle (DMSO) or 10 *μ*M pevonedistat. (**b**) Lysates from cells that received a toxic dose of 200 ng/ml TNF-*α* or 5 ng/ml TNF-*α*+10 *μ*M pevonedistat were western blotted for the indicated proteins. An asterisk indicates cross reactivity to the protein ladder in the caspase-8 (p18) blot. (**c**) Activation of caspase-8 was quantified by an *in vitro* caspase assay for the cleavage of a fluorometric substrate. All viability experiments were performed in triplicate, and error bars indicate±S.E.M. (**d**) The activation state of caspase-8 was determined after 6 h of treatment with the indicated compounds. Extracts were created with lysis buffers that contained either 1% Triton X-100 or 1% SDS. (**e**) Cells were treated with 200 ng/ml TNF-*α* in combination with pevonedistat±epoxomicin. Extracts were western blotted with antibodies specific for either the p10 (top) or p18 (middle) subunits of caspase-8. The presumed caspase-8 subunits are indicated to the left of the image based on the expected size of the product. Approximate molecular sizes of proteins (in kDa) are given to the right of blots.

To characterize how pevonedistat potentiated cytotoxic caspase-8 and TNF signaling, cells were treated with a broad range of TNF-*α* concentrations for 8 h. Cells that tolerated TNF-*α* single-agent treatment (LC_50_=454.3 ng/ml) were approximately 50-fold more sensitive to TNF-*α* (LC_50_=8.0 ng/ml) when concurrently dosed with pevonedistat (Figure 4a), and this sensitization was still apparent after 24 h (Supplementary Figure S3a). The toxicity of the combination treatment of 5 ng/ml TNF-*α*+pevonedistat was determined to be approximately equal to the toxicity of 200 ng/ml TNF-*α* single-agent treatment at 8 h (20–30% death) and at 24 h (>95% death). These two treatments were then directly compared over time. Western blotting of lysates identified a similar pattern of caspase-8 and caspase-3 cleavage within 2–4 h (Figure 4b). However, after 6 h, there were approximately 6-fold more p10 staining in the pevonedistat+TNF-*α*-treated lysates than from single-agent TNF-*α* treatment (Figure 4b, arrow). Interestingly, the relative amounts of other subunits, such as p18 (arrowhead), were essentially the same between treatment regimens. Both treatments also resulted in a caspase-8-specific cleavage^33^ of the pro-survival protein cFLIP-L into the p43 fragment, but the loss of NEDD8-cullin staining only occurred with pevonedistat. The combination of 5 ng/ml TNF-*α*+pevonedistat resulted in ~2-fold higher caspase-8 activity than single-agent 200 ng/ml TNF-*α* (Figure 4c). However, caspase-8 activity did not significantly differ between pevonedistat treatment combined with either 5 or 200 ng/ml TNF-*α*.

Caspase-8 ubiquitination leads to protein inactivation^34^ or degradation,^35^ but under specific conditions it instead leads to protein activation.^36^ This ubiquitin-mediated activation of caspase-8 is regulated by the CRL family member cullin-3 and requires the ubiquitination of a highly onserved lysine within the p10 domain of the protein.^36^ To determine whether ubiquitination of caspase-8 drives the pevonedistat+TNF-*α* toxicity, cells were treated for 6 h to capture the ubiquitination state of caspase-8 immediately before widespread cell death previously observed at 8 h. Extracts were created with an SDS-based lysis buffer, in addition to a Triton X-100 buffer, because SDS was shown to enhance the detection of ubiquitinated caspase-8.^36^ Western blotting with two caspase-8 antibodies specific to the p10 domain did not identify protein banding indicative of caspase-8 polyubiquitination but again demonstrated the accumulation of p10 after pevonedistat+TNF-*α* (Figure 4d and Supplementary Figure S3b). Treatment with a proteasome inhibitor (epoxomicin) in addition to TNF-*α*± pevonedistat also did not result in caspase-8 ubiquitination (Figure 4e). Proteasome inhibition resulted in a slight accumulation of p18, but not p10, which is consistent with a previous observation.^37^ The role of cullin-3 in pevonedistat+TNF-*α* toxicity was then assessed. It was hypothesized that if cullin-3 mediated the toxicity, then siRNA knockdown of the protein would mimic the pharmacological effect of pevonedistat inhibition and make cells sensitive to TNF-*α*. However, knockdown of cullin-3 expression actually limited cell death caused by either pevonedistat+TNF-*α* or a high dose of single-agent TNF-*α* (Supplementary Figure S4). Thus, the role of caspase-8 ubiquitination in the pevonedistat+TNF-*α* toxicity remains unclear.

### Pevonedistat and TNF-*α* activate necroptosis when caspases are inhibited

**Figure 5:**
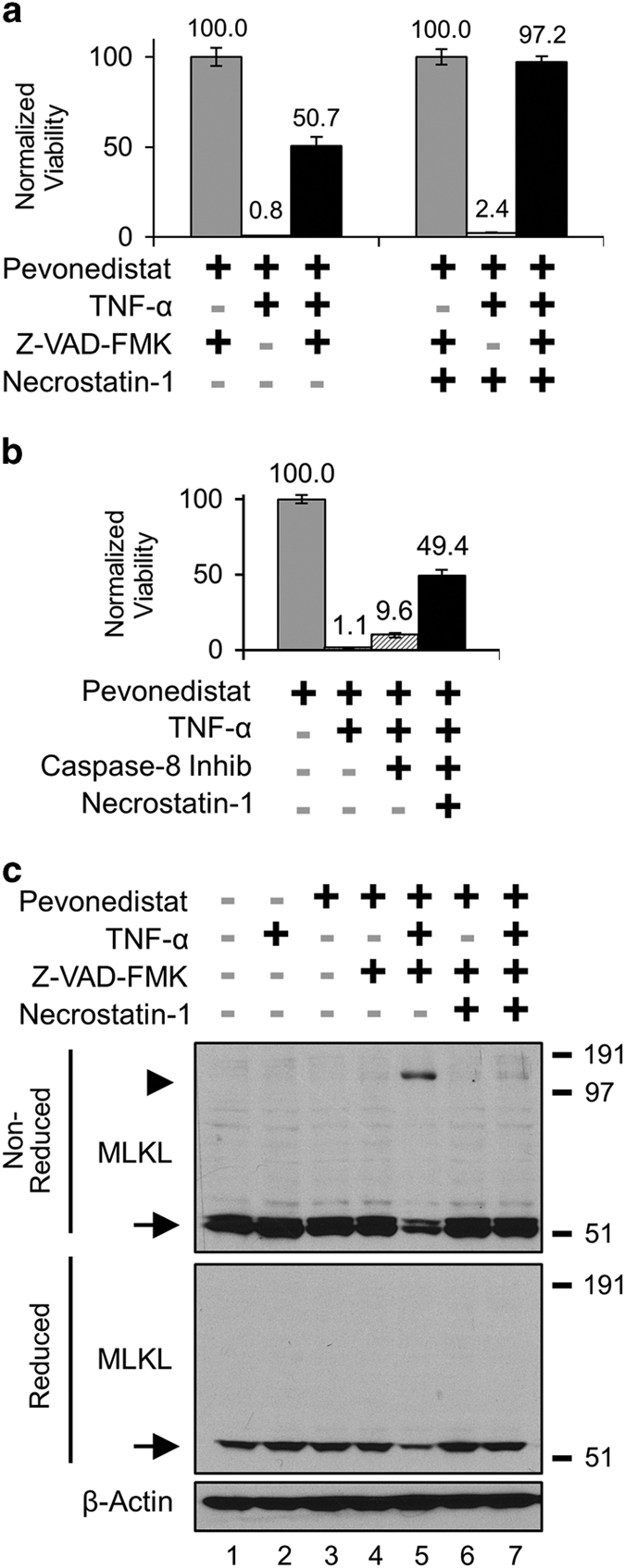
Pevonedistat+TNF-*α* activated necroptosis when apoptosis was inhibited. (**a**) H-4-II-E cells were treated for 48 h with compounds to modulate apoptosis and necroptosis. The indicated treatments included (left) the pan-caspase inhibitor Z-VAD-FMK (20 *μ*M); and (right) the RIP1 inhibitor Necrostatin-1 (50 *μ*M). (**b**) Cells received 20 *μ*M of the caspase-8 inhibitor Z-IETD-FMK or the indicated compounds for 48 h. All viability experiments were performed in triplicate, and error bars indicate±S.E.M. (**c**) Extracts from cells treated for 24 h were western blotted under either non-reduced (upper) or reduced (lower) conditions. The molecular sizes corresponding to approximately 53 kDa (arrow) and 150 kDa (arrowhead) are indicated. Molecular size markers from a protein standard are indicated to the right of blots.

Necroptosis is an alternative form of cell death distinct from apoptosis that can occur when caspase-8 remains in its inactive form.^38^ To demonstrate that H-4-II-E cells can activate necroptosis when treated with an established combination^39,40^ of cycloheximide, TNF-*α*, and caspase inhibitors (Supplementary Figure S5a). In 48-h treatments with pevonedistat+TNF-*α*, most cells died (~1% viability), but survival was boosted to ~50% with the inclusion of the apoptosis/pan-caspase inhibitor Z-VAD-FMK (Figure 5a, left). Cell death was prevented (97% viability) when Z-VAD-FMK was dosed in combination with the necroptosis inhibitor Necrostatin-1 (Figure 5a, right). Individual caspase inhibitors were used to understand their contribution to apoptosis/necroptosis. The caspase-8 inhibitor (Z-IETD-FMK) prevented ~10% of cell death caused by pevonedistat+TNF-*α*, while the treatment with Necrostatin-1 in addition to Z-IETD-FMK boosted survival to approximately 50% (Figure 5b). Inhibitors of caspases 3/7, 6, and 9 were all less successful at preventing cell death than caspase-8 inhibition (Supplementary Figure S5b).

An alternative method to identify necroptosis is to western blot for trimerized MLKL in non-reduced cell lysates.^30^ As determined by the onset of cell death, necroptosis was activated between 24 and 48 h of treatment (Supplementary Figure S6a). In cells treated for 24 h, the approximately 150 kDa trimeric MLKL band (Figure 5c, arrowhead) was detected only after treatment with pevonedistat+TNF-*α*+Z-VAD-FMK (Lane 5). The 53-kDa MLKL monomer was detected in lysates (Figure 5c, arrows) in both non-reduced (upper) and reduced (lower) blots. Necrostatin-1 also prevented the formation of the MLKL trimer (Lane 7). Western blots with a second MLKL antibody confirmed these results (Supplementary Figure S6b). Pevonedistat+TNF-*α* did not cause MLKL trimerization at 6 h, a time point preceding widespread cell death (Supplementary Figure S6c). These results demonstrate that, when apoptosis was blocked, pevonedistat+TNF-*α* activated necroptosis.

### Pevonedistat and TNF-*α* synergistically cause liver damage in rats

**Figure 6:**
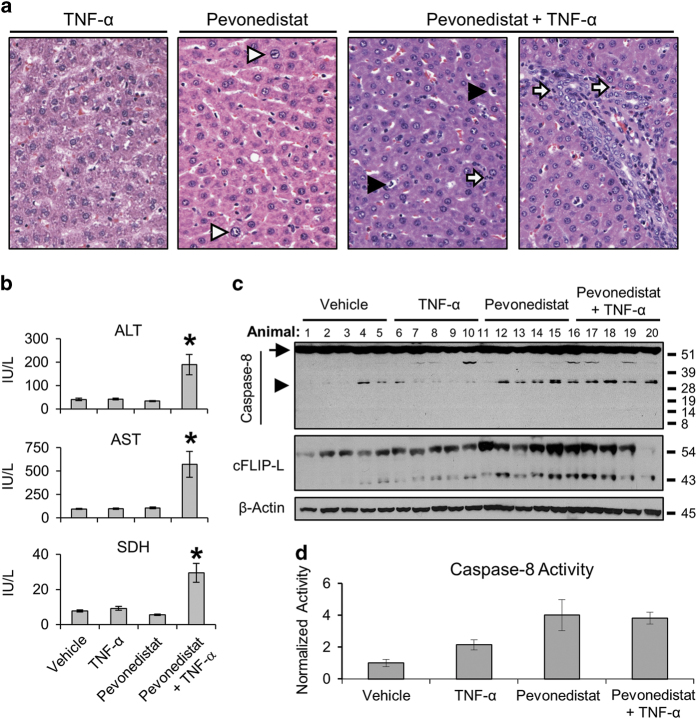
Combination of pevonedistat and elevated TNF-*α* is toxic to rats. Sprague-Dawley rats (*n*=8) were administered single doses of vehicle, TNF-*α*, pevonedistat, or pevonedistat+TNF-*α*. Scheduled necropsy occurred 24 h later. (**a**) Representative microscopic images of H&E-stained livers from animals that received the indicated treatments: hepatocyte karyomegaly (white arrowhead), single-cell necrosis (black arrowhead), and neutrophilic infiltration (white arrow). (**b**) Rats were administered single doses of the indicated compounds; markers of liver injury were analyzed approximately 24 h later. Serum was available for only six animals that received pevonedistat+TNF-*α*. The group-mean concentrations ALT, AST, and SDH are indicated. Asterisks indicate statistically significant (*P*<0.01) differences. (**c**) Livers from 20 rats that received the indicated compounds were western blotted for caspase-8 and cFLIP-L expression. Full-length pro-caspase-8 (arrow) and the cleaved p32 subunit (arrowhead) are indicated. Approximate molecular sizes of proteins (in kDa) are given to the right of blots. (**d**) Tissue liver extracts from the same 20 rats were used in a caspase-8 activity assay. Each sample was analyzed in triplicate, values averaged, and normalized against the group mean value for vehicle control. Error bars indicate±S.E.M.

The *in vivo* effects of pevonedistat and TNF-*α* were assessed in Sprague-Dawley rats. The dose of pevonedistat administered to rats was known from previous investigations to be well tolerated, and the dose of recombinant rat TNF-*α* activated TNF signaling without toxic side effects.^4^ Animals within each group (*n*=8) first received either vehicle or 10 *μ*g/kg TNF-*α*, followed by either a second vehicle or 120 mg/kg pevonedistat 1 h later. Two animals dosed with the combination treatment exhibited moribund conditions and were euthanized within 10 h. There was a clear difference in liver damage of single-agent *versus* combination treatments in rats. The incidence and severity of microscopic liver findings for five representative animals from each dose group are presented in Table 1. The livers of animals dosed with pevonedistat+TNF-*α* had minimal-to-mild single-cell necrosis and neutrophilic infiltration. Representative histological images in Figure 6a illustrate karyomegaly (white arrowhead) in the livers from animals that received pevonedistat alone and necrosis (black arrowhead) and neutrophilic infiltrate (white arrow) in the combination-treated livers. Animals that received the combination treatment had significant ~5-fold elevation of the serum markers alanine transaminase (ALT), aspartate transaminase (AST) and sorbitol dehydrogenase (SDH) compared with those that received single-agent treatments (Figure 6b). Western blotting of liver extracts identified uncleaved caspase-8 (Figure 6c, arrow) in all animals and the p32 fragment of caspase-8 was observed in 9/10 animals that received pevonedistat±TNF-*α* (arrowhead). Neither p10 nor p18 (data not shown) were detected. Staining of the cleaved cFLIP-L 43-kDa fragment was strongest in samples that also had caspase-8 cleavage. There was a 4-fold elevation of caspase-8 activity in the pevonedistat±TNF-*α* groups compared with vehicle (Figure 6d). Whether caspase-8 activation was the principle driver of toxicity in rats could not be established.

## Discussion

In this study, we have identified a novel *in vitro* and *in vivo* synergistic cytotoxicity between the NAE inhibitor pevonedistat and the pro-inflammatory cytokine TNF-*α*. Pevonedistat sensitized cells to TNF-*α* and activated apoptosis at otherwise tolerated TNF-*α* concentrations. Cell death was mediated by caspase-8, and pevonedistat+TNF-*α* treatment resulted in the accumulation of the caspase-8 p10 protease. These results have led to an improved understanding of potential clinical pevonedistat toxicities that can occur in patients with a preexisting pro-inflammatory state.^5^ Patient inclusion/exclusion criteria were established in clinical trials with pevonedistat to exclude those who have active uncontrolled infections^6–10^ or have recently received antibiotics.^8–10^

Inhibition of the NEDD8 pathway was achieved through pevonedistat treatment (Figure 1b) or siRNA knockdown of NEDD8 expression (Supplementary Figure S1). Cell death caused by pevonedistat+TNF-*α* is not a result of an off-target effect of pevonedistat, because the NEDD8 knockdown also conferred sensitivity to TNF-*α*. Conversely, knockdown of CDT1 expression and FACS analysis were used to demonstrate that cell-cycle arrest did not drive the pevonedistat+TNF-*α* toxicity (Figure 2). Although the majority of the data discussed herein utilized a rat hepatoma H-4-II-E cell line to characterize the synergistic toxicity, this was reflected in animal studies as well. Pevonedistat caused karyomegaly in rats, likely due to the drug mechanism of action, but hepatocyte necrosis was only observed in the combination treatment (Table 1). Thus it is clear that pevonedistat potentiates cytotoxic TNF-*α* signaling.

Pevonedistat lowered the activation threshold for TNF-mediated cell death by conferring sensitivity to low concentrations of TNF-*α*. This result was demonstrated through viability assays and western blottings for markers of cell death. After 8 h of treatment, cells were approximately 50-fold more sensitive to pevonedistat+TNF-*α* than single-agent treatment (Figure 4a). Additional *in vitro* experiments confirmed that pevonedistat+TNF-*α* killed exclusively through apoptosis. Activation of necroptosis only occurred when apoptosis was disabled (Figure 5a). Detection of trimeric MLKL in non-reduced lysates validated this biomarker of necroptosis (Figure 5c and Supplementary Figure S6b). These findings are consistent with another study which demonstrated that under certain conditions pevonedistat activated necroptosis.^41^

Caspase-8 was clearly the mediator of the pevonedistat+TNF-*α* synergistic cytotoxicity as expression knockdown (Figure 3e) or chemical inhibition (Figure 5b) prevented cell death. The kinetics of caspase-8 activation were assessed by western blotting. A high dose of TNF-*α* resulted in a low continuous level of caspase-8 processing while pevonedistat+TNF-*α* treatment caused a rapid cleavage of the protein between 4 and 8 h (Figure 4b). By 8 h, the relative amount of caspase-8 activation was nearly identical between pevonedistat dosed in combination with 5 or 200 ng/ml TNF-*α* (Figures 4c and d). Notably, the pevonedistat+TNF-*α* combination caused a 6-fold accumulation of the p10 protease compared with single-agent TNF-*α* (Figures 4b and d). Proteasome inhibition had no effect on p10 levels after pevonedistat+TNF-*α* treatment (Figure 4e), so this accumulation appears because of increased processing and not simply decreased degradation. Although p10 can affect mitochondria function, little is known about the mechanism of its stabilization/turnover.^24,37^ The accumulation of p10 in these experiments appears to be novel finding and establishes pevonedistat as a tool compound for investigating caspase-8 regulation.

The results of this study demonstrate that the combination of the NAE inhibitor pevonedistat and the pro-inflammatory cytokine TNF-*α* is toxic. The driver of *in vitro* toxicity appears to be enhanced cleavage/activation of the caspase-8 p10 protease, which in turn activated apoptosis. However, the molecular mechanism that links pevonedistat to caspase-8 remains unclear in the pevonedistat and TNF-*α* cytotoxicity model. As cullin-3 can ubiquitinate caspase-8 (Jin *et al.* ^36^) and is also inhibited by pevonedistat, it was an obvious candidate for investigation, but cullin-3 knockdown did not increase sensitivity to single-agent TNF-*α* (Supplementary Figure S4). Ultimately, a role for cullin-3 in mediating the synergistic toxicity was not established. Single-agent pevonedistat is known to stabilize the expression of ≥120 different proteins,^42^ none of which are known to interact with caspase-8. A higher-throughput approach is needed to determine if any unrecognized proteins become stabilized in response to pevonedistat+TNF stimulation. Further investigations using pevonedistat as a tool compound will lead to a better understanding of the molecular mechanisms that underlie programmed cell death.

## Materials and Methods

### Reagents

Pevonedistat was synthesized by Millennium Pharmaceuticals, Inc. The following reagents were purchased from their respective companies: recombinant rat TNF-*α* (Peprotech, Rocky Hill, NJ, USA); caspase inhibitors Z-VAD-FMK and Z-IETD-FMK (R&D Systems, Minneapolis, MN, USA); Necrostatin-1 (Sigma-Aldrich, St. Louis, MO, USA); and Epoxomicin (Sigma-Aldrich). Antisera were purchased from the following companies: *β*-Actin, cleaved caspase-3, cleaved caspase-8 (p18), cFLIP, cullin-3, I*κ*B*α*, phospho-I*κ*B*α*, NEDD8, PARP, pro-caspase-3, pro-caspase-6, pro-caspase-7, pro-caspase-8 (p10), pro-caspase-9 (Cell Signaling, Danvers, MA, USA); MLKL (Millipore, Billerica, MA, USA); CDT1 (Santa Cruz Biotechnology, Dallas, TX, USA); and BID (eBioscience, San Diego, CA, USA). Complete antisera details are provided in Supplementary Information.

### Cell culture

The rat hepatoma H-4-II-E cell line was selected to model pevonedistat toxicities because of its common use in the assessment of toxic compounds.^43,44^ H-4-II-E cells were purchased from American Type Culture Collection (Manassas, VA, USA) and were cultured following the manufacturer’s instructions. Briefly, cells were cultured in MEM (Life Technologies, Carlsbad, CA, USA) supplemented with 10% FBS (American Type Culture Collection) and incubated at 37 °C with 5% CO_2_. For routine culture, cells were supplemented with 10 U/l of penicillin and 10 ug/l of streptomycin (Life Technologies). For passaging, cells were washed once with PBS (Life Technologies), treated with 0.05% trypsin-EDTA (Life Technologies), supplemented with fresh media, and pelleted in a clinical centrifuge.

### Cell-based assays

Intracellular ATP was quantified in 96-well plates with CellTiter-Glo (Promega, Madison, WI, USA) following the manufacturer’s instructions. After 30 min, luminescence was quantified using a Victor X3 Plate Reader (Perkin-Elmer, Waltham, MA, USA). Data were analyzed in Prism 5 (Graphpad Software, La Jolla, CA, USA), and the least-squares method was used to determine the concentraton causing lethality for 50% of cells (LC_50_). For caspase activity assays, cells were treated in 60-mm tissue culture dishes, removed with trypsin-EDTA, and plated at 50 000 cells/well within a 96-well plate. Caspase-8 activities were determined with Caspase-Glo (Promega) following the manufacturer’s instructions, and luminescence was quantified using a Victor X3 Plate Reader. For TUNEL assays, cells were plated at confluence on glass coverslips and treated for 6 h. Apoptotic cells were detected with DeadEnd Colormetric TUNEL assay (Promega), following the manufacturer’s instructions.

### Lysate preparation and western blotting

Cultured cells were washed twice with ice-cold PBS and lysed in plates with 1× Cell Lysis Buffer (Cell Signaling). Cells were scraped from the dish, transferred to a 2.0-ml tube, sonicated for 20 s using a Virsonic Ultrasonic Cell Disruptor 1000 (VirTis, Gardiner, NY, USA), and centrifuged at a maximum speed for 10 min at 4 °C. The supernatant was reserved, and protein concentration was determined using a Bradford assay (Bio-Rad, Hercules, CA, USA). The Triton X-100 and SDS cell lysis buffers were prepared as previously described.^36^ For routine western blotting, 10–25 *μ*g of lysate was mixed with 1× NuPage LDS Sample Buffer (Life Technologies) and 1× NuPage Reducing Agent (Life Technologies). Cell lysates were boiled at 100 °C for 10 min and loaded into 4–12% NuPage Bis-Tris gels (Life Technologies), and proteins were separated by electrophoresis. The NuPage Reducing Agent and heating step were not used for non-reducing western blots. Proteins were then transferred onto 0.2-*μ*m pore size nitrocellulose membranes (Life Technologies) and incubated with primary and secondary antibodies. Where indicated, protein band intensity was quantified using ImageJ 1.34s.^45^

### FACS analysis

DNA nuclear content was determined as previously described.^15^ Actively dividing H-4-II-E cells were treated with pevonedistat and/or TNF-*α* for 8 h. Before the end of treatment, cells were spiked with 10 *μ*M bromodeoxyuridine (Brd-U) (BD Pharmingen, San Diego, CA, USA). After 30 min, cells were fixed in ethanol, incubated with a FITC–anti-Brd-U secondary antibody (BD Pharmingen), and then incubated with 10 *μ*g/ml propidium iodide (PI) (BD Pharmingen). Labeled cells were measured for Brd-U and PI staining on a FACSCalibur flow cytometer (Benton Dickinson, Franklin Lakes, NJ, USA). Cell cycle data were analyzed using FACSDiva (v 6.1.1) (BD Biosciences, Franklin Lakes, NJ, USA).

### siRNA knockdown

H-4-II-E cells were transfected with either a non-targeting control pool of siRNAs or with individual siGenome siRNA oligonucleotide duplexes (Dharmacon, Lafayette, CO, USA) designed to silence target rat genes *caspase-8* and *cdt1.* Cells were plated sparsely (10 000 cells/well in 96-well plates and 500 000 cells/well in a six-well tissue-culture plate) in antibiotic-free media. The following day, cells were transfected with 25 nM of siRNAs using Lipofectamine RNAiMAX (Life Technologies) for 72 h. Following transfection, cells were treated with pevonedistat and/or TNF-*α* for 24–48 h. Successful knockdown were verified by western blotting. Sequences for siRNAs used in experiments are included in Supplementary Information.

### *In vivo* rat model

All animal experiments for this study were conducted in accordance with Millennium Pharmaceuticals, Inc. Institutional Animal Care and Use Committee Guidelines. Ten-week-old male Sprague-Dawley rats were purchased from Charles River Laboratories (Raleigh, VA, USA). Across two studies, a total of eight animals in each group were dosed with vehicle, TNF-*α*, pevonedistat, or pevonedistat+TNF-*α*. Animals were first intravenously administered either vehicle (1× PBS) or 10 *μ*g/kg TNF-*α*. One hour later, they were subcutaneously administered vehicle (20% sulfobutyl ether beta-cyclodextrin in 50 mM citrate buffer, pH 3.3) or 120 mg/kg pevonedistat. Scheduled euthanasia occurred 24 h postdose. Unscheduled euthanasia was performed when animals exhibited moribund conditions. Serum was collected at necropsy and analyzed by Idexx Laboratories (North Grafton, MA, USA) for serum chemistry markers of liver damage (ALT, AST, and SDH). Additionally, the livers from five animals in each group were removed, separated into two sections and either frozen at −80 °C for subsequent protein analysis or fixed in 10% neutral buffered formalin, embedded in paraffin, sectioned at 4–6 *μ*m, mounted on glass slides, stained with hematoxylin and eosin, and analyzed with an Olympus BX51 light microscope (Tokyo, Japan) for histopathology assessment. Microscopic findings were recorded in concordance with the standardized nomenclature for classifying lesions within the livers of rats.^46^

## Figures and Tables

**Table 1:** Incidence and severity of liver microscopic findings

*Test article*	*Vehicle*	*TNF-α*	*Pevonedistat*	*Pevonedistat+TNF-α*
*Incidence (n=5)*				
Single-cell necrosis (periportal)	0	0	0	4
Neutrophilic infiltration (periportal)	0	0	0	4
Hepatocyte karyomegaly	0	0	4	5
				
*Mean severity (Graded 0–4)*				
Single-cell necrosis (periportal)	0	0	0	1.4
Neutrophilic infiltration (periportal)	0	0	0	0.8
Hepatocyte karyomegaly	0	0	0.8	1.0

Five representative Sprague-Dawley rats were administered single doses of the indicated compounds; livers were analyzed at necropsy by histopathology. The incidence of microscopic findings and average severity, in brackets, of the findings are indicated. Findings were graded on a scale of 0 (absent), 1 (minimal), 2 (mild), 3 (moderate), or 4 (marked).
