# Supplementary material for: The NAE inhibitor pevonedistat (MLN4924) synergizes with TNF-α to activate apoptosis
Source: Cell Death Discov. 2015 Oct 5;1:15034–. doi: 10.1038/cddiscovery.2015.34 (PMC4979425; doi:10.1038/cddiscovery.2015.34)
Supplement: Supplementary Information [file cddiscovery201534-s1.doc]

# Online Supplementary Information

# Title: The NAE Inhibitor Pevonedistat (MLN4924) Synergizes With TNF-α to Activate Apoptosis

# Supplementary Figure Legends

**Supplementary Figure S1. siRNA knockdown of NEDD8 in H-4-II-E cells confers sensitivity to TNF-α.** (**a**) H-4-II-E cells were transfected with either a siRNA pool against a non-targeting control or a single siRNAs against NEDD8. Cell lysates were collected 4 days later and Western blotted for the indicated proteins. NEDD8-cullin (arrowhead) and free NEDD8 (arrow) are shown with approximate molecular-size markers (in kDa) to the right. Lysates were also Western blotted for phosph‑IκBα to determine which siRNA oligonucleotide resulted in the greatest amount of NEDD8/cullin/proteasome pathway inhibition. (**b**) Knockdown of NEDD8 in H-4-II-E cells conferred sensitivity to TNF-α. Cells were transfected with control or NEDD8-A siRNA. Four days later cells were treated with the indicated concentrations of TNF-α. Cell viability was determined after 24 hours by CellTiter‑Glo® assay. Viability experiments were performed in triplicate and error bars indicate ± Standard Error of the Mean (SEM). ** indicate statistically significant (p < 0.01) differences.

**Supplementary Figure S2. Multiple cell types are sensitive to pevonedistat + TNF-α.**

The median lethal concentration (LC_50_) was determined using the least squares method. Solid lines indicate a non-linear fit of the data. For Supplementary Figure S2A and Supplementary Figure S2B, cell viabilities were determined with a WST-8 assay (Enzo Life Sciences). (**a**) Primary rat hepatocytes treated with pevonedistat in combination with either PBS (red) or recombinant rat TNF-α (100 ng/ml; blue) for 48 hours. Cell viability was determined with a WST-8 assay and normalized to DMSO controls. (**b**) Primary rat liver Kupffer cells were treated with pevonedistat in combination with either PBS (red) or recombinant rat TNF-α (100 ng/ml; blue) for 48 hours. For Supplementary Figures S2c to S2f, cell viabilities were determined by a CellTiter-Glo® intracellular ATP assay. (**c**) Rat renal proximal tubular NRK-52E cells were treated with pevonedistat in combination with either PBS (red) or recombinant rat TNF-α (100 ng/ml; blue) for 48 hours. (**d**) Human leukemic monocyte THP-1 cells were treated pevonedistat in combination with either PBS (red) or recombinant human TNF-α (100 ng/ml; blue) for 24 hours. (**e**) Human leukemic monocyte THP-1 cells were treated with pevonedistat in combination with either PBS (red) or an agonist antibody to the TNF-receptor (TNF‑R) (1 μg/ml; green) for 24 hours. (**f**) Human liver carcinoma HEP-G2 cells were treated with pevonedistat in combination with either PBS (red) or an agonist antibody to TNF-R (1 μg/ml; green) for 48 hours. Error bars indicate ± SEM.

**Supplementary Figure S3. 200 ng/ml TNF-α and 5 ng/ml TNF-α + 10 μM pevonedistat is cytotoxic.** (**a**) H-4-II-E cells were treated for 24 hours with a range of TNF-α concentrations (0.1 to 500 ng/ml) in combination with either DMSO or 10 μM pevonedistat. Cell viabilities were determined by a CellTiter-Glo® assay. Viability experiments were performed in triplicate and error bars indicate ± SEM. (**b**) Pro-caspase-8 and the p10 subunit were not polyubiquitinated by pevonedistat + TNF-α. Cells were treated with 5 or 200 ng/ml TNF-α ±10 μM pevonedistat for 6 hours. Cell extracts were created using lysis buffers that contained either 1% Triton X-100 or SDS, as described for Figure 4d. Lysates were Western blotted for caspase-8 with an antibody directed against the C‑terminal portion of the protein (Abcam). Caspase-8 migrates at 55‑kDa, and an asterisk indicates a non-specific band that migrates slightly larger (approximately 60 kDa).

**Supplementary Figure S4. Cullin-3 knockdown decreased sensitivity to treatment with pevonedistat + 5 ng/ml TNF-α or 200 ng/ml TNF-α.** (**a**) H-4-II-E cells were transfected with either a siRNA pool against a non-targeting control or a single siRNA against cullin-3. Cell lysates were collected 4 days later and Western blotted for the indicated proteins. Western blotting for cullin-3 confirmed nearly complete knockdown of the protein, with the expected increase in NRF2 expression. (**a**) Knockdown of cullin-3 delayed, but did not prevent, cell death caused by pevonedistat + TNF-α. Cells were transfected with control or cullin-3 siRNA and treated with 10 μM pevonedistat + 5 ng/ml TNF-α. Cell viability was determined after 8, 12, 24 and 48 hours by CellTiter‑Glo® assay. (**c**) Knockdown of cullin-3 in cells conferred less sensitivity to 200 ng/ml TNF-α. Cells were transfected with control, cullin-3, or caspase-8-A (the same method as the experiment presented in Figure 3D). Four days later, cells received 200 ng/ml TNF-α. Cell viability was determined after 6 and 24 hours by CellTiter‑Glo® assay. Caspase-8 knockdown served as a positive control. Viability experiments were performed in triplicate and error bars indicate ± SEM.

**Supplementary Figure S5. Necroptosis can be activated in H-4-II-E cells.** (**a**) Cycloheximide was used to drive necroptosis in the H-4-II-E-cultured cell line. Cells were treated with 2‑μg/ml cycloheximide in combination with 5 ng/ml TNF-α, 20 μM Z-VAD-FMK or 50 μM necrostatin-1 for 48 hours. (Left) The indicated treatments included the pan-caspase inhibitor Z-VAD-FMK (Left). The indicated treatments additionally received the RIP1 inhibitor necrostatin-1 (Right). Cell viability was determined by CellTiter‑Glo® assay. Numbers above bars indicate the normalized cell viability. (**b**) Cells received the following treatments: 10 μM pevonedistat single agent (green bars); pevonedistat + 5 ng/ml TNF-α (purple bars); pevonedistat + TNF-α + 20 μM of the indicated caspase inhibitor (red bars), or pevonedistat + TNF-α + caspase inhibitor + 50 μM necrostatin-1 (blue bars). Z‑DEVD-FMK is an inhibitor of caspase-3/7, Z-VEID-FMK is an inhibitor of caspase-6, Z-IETD-FMK is an inhibitor of caspase-8, and Z-LEHD-FMK is an inhibitor of caspase-9. Cell viability was determined by CellTiter‑Glo® assay after 48 hours of treatment. Numbers above bars indicate the normalized cell viability. Viability experiments were performed in triplicate and error bars indicate ± SEM.

**Supplementary Figure S6. Necroptosis is activated in H-4-II-E cells treated with pevonedistat + TNF-α + Z-VAD-FMK.** (**a**) Cells received the following treatments: 10 μM pevonedistat single agent + 5 ng/ml TNF-α (red bars); pevonedistat + TNF-α + 20 μM Z-VAD-FMK (blue bars); pevonedistat + TNF-α + necrostatin-1 (green bars); or pevonedistat + TNF-α + Z-VAD-FMK + necrostatin-1 (purple bars). Cell viabilities were determined by CellTiter‑Glo® assay at 4, 8, 16, 24 and 48 hours of treatment. Values were normalized to pevonedistat single agent. Viability experiments were performed in triplicate and error bars indicate ± SEM (**b**) Cells received the indicated treatments for 24 hours. The same extracts used from the experiment presented in Figure 4C were Western blotted under non-reduced (upper) or reduced (lower) conditions. The molecular sizes corresponding to approximately 53 kDa (arrow) and 150 kDa (arrowhead) are indicated. The MLKL antibody used was produced by Abcam (Catalog # ab172868). (**c**) Cells were treated for 6 hours with the indicated compounds. Extracts were Western blotted under reduced or non-reduced conditions. The molecular size corresponding to approximately 53 kDa (arrow) is indicated. The MLKL antibody used was produced by Millipore (Catalog # MABC604). Molecular-size markers from a protein standard (in kDa) are indicated to the right.

# Supplementary Methods

**Reagents**

The following reagents were purchased from their respective companies: recombinant human TNF-α (Cell Signaling Technologies); cycloheximide (FisherScientific); and caspase inhibitors Z-DEVD-FMK, Z-VEID-FMK, Z-IETD-FMK, Z-LEHD-FMK (R&D Systems).

**Cell-Based Assays**

Cell viability of primary cells was determined by reduction of WST-8 (Enzo Life Sciences). All other viability assays used CellTiter-Glo® (Promega) to determine intracellular ATP.

**Cell Culture**

Cryopreserved platable primary rat hepatocytes and liver Kupffer cells were purchased from Life Technologies and cultured according to manufacturer’s instructions. Media and treatments were replaced daily. The rat proximal tubule line NRK-52E, the human acute monocytic leukemia THP-1 line, and the human hepatocellular carcinoma line HEP-G2 were purchased from American Type Culture Collection and were cultured following manufacturer’s instructions. All media was supplied from Life Technologies and supplemented with appropriate maintenance cocktails. Primary rat hepatocytes were cultured in William’s E Medium, Kupffer cells were cultured in Advanced DMEM Medium, NRK-52E cells were cultured in DMEM, THP-1 cells were cultured in RMPI 1640 Medium, and HEP-G2 cells were cultured in EMEM. Cells were supplemented with 10% FBS (Life Technologies) and incubated at 37°C with 5% CO_2_. NRK‑52E, THP-1, and HEP-G2 cells were supplemented with 10,000 U/ml of penicillin and 10,000 μg/ml of streptomycin (Life Technologies).

**siRNA Knockdown**

Details for siRNA knockdown of target genes can be found in the main text. Reagents were purchased from Dharmacon. A pooled mix of 4−non-targeting siRNA molecules (siGENOME Non-Targeting siRNA Pool#1, Catalog # D-001206-13-20) were used as a negative control. The sequences of oligonucleotide duplexes (5′ to 3′) that were used in these experiments are: Caspase-8-A, GAACGAUCAAGCACAGAGA; Caspase-8-B, GAGGAUUCAUCAUCUUACA; Caspase-8-C, GCAGAAAACAACUUGGUUA; Caspase‑8‑D, UGAGAUCCCUAAAUGUAAA; CDT1-A, GCUCGUGGCUCCUGAGUUC; Cullin-3-A, GGCCACAUAUUUACAGUUA; NEDD8-A, UUACAAGAUUCUAGGUGGU; NEDD8-B, GAUUGAGUUGACAUCGAA; NEDD8-C, CAGCAAGGUGGAACGAAU; and NEDD8-D, CAUCUACAGUGGCAAACAA.

**Detailed Antibody Information**

| **Antibody Name** | **Vendor** | **Catalog #** | **Dilution** | **Primary Buffer** |
| --- | --- | --- | --- | --- |
| β-Actin | CST | 4967 | 3000 | BSA |
| BID | EBioscience | 14-5944 | 1000 | Milk |
| CDT1 | Santa Cruz | sc-28262 | 1000 | BSA/Milk |
| Clv Caspase-3 | CST | 9664 | 1000 | Milk |
| Clv Caspase-8 (p18) | CST | 9429 | 250-1000 | BSA |
| cFLIP | CST | 8510 | 500-1000 | BSA |
| Cullin-3 | CST | 2759 | 1000 | BSA |
| HRP-anti-Rabbit | CST | 7074 | 1000 | Milk |
| HRP-anti-Mouse | CST | 7076 | 1000 | Milk |
| HRP-anti-Rat | CST | 7077 | 1000 | Milk |
| IκBα | CST | 4814 | 1000 | Milk |
| phospho-IκBα | CST | 9246 | 1000 | Milk |
| MLKL | Millipore | MABC604 | 4000 | NGS/Milk |
| MLKL | Abcam | ab172868 | 1500 | BSA/Milk |
| NEDD8 | CST | 2754 | 1000 | BSA |
| NRF2 | CST | 8882 | 1000 | BSA |
| PARP | CST | 9542 | 1000 | Milk |
| Pro-Caspase-3 | CST | 9665 | 1000 | Milk |
| Pro-Caspase-6 | CST | 9762 | 1000 | Milk |
| Pro-Caspase-7 | CST | 9492 | 1000 | Milk |
| Pro-Caspase-8 (p10) | CST | 4790 | 250-1000 | BSA |
| Pro-Caspase-8 (p10) | Abcam | ab138485 | 2000 | Milk |
| Pro-Caspase-9 | CST | 9508 | 1000 | Milk |
| TNF-R | Santa Cruz | sc-8436 | ( - ) | ( - ) |
|  |  |  |  |  |

**( - ) =** not applicable; **BSA** = 5% w/v bovine serum albumin, 0.1% Tween-20, 1× PBS; **BSA/Milk** = 5% w/v bovine serum albumin, 5% w/v non-fat dry milk, 0.1% Tween-20, 1× PBS; **CST** = Cell Signaling Technologies; **HRP** = horseradish peroxidase; **Milk** = 5% w/v non-fat dry milk, 0.1% Tween-20, 1× PBS; **NGS/Milk** = 5% v/v normal goat serum, 5% w/v non-fat dry milk, 0.1% Tween-20, 1× PBS.
